# Training Inhibition and Social Cognition in the Classrooms

**DOI:** 10.3389/fpsyg.2020.01974

**Published:** 2020-08-21

**Authors:** Nastasya Honoré, Marine Houssa, Alexandra Volckaert, Marie-Pascale Noël, Nathalie Nader-Grosbois

**Affiliations:** Psychological Sciences Research Institute, UCLouvain, Louvain-la-Neuve, Belgium

**Keywords:** executive functions, inhibition, social cognition, Theory of Mind, social information processing, training

## Abstract

Executive functions and social cognition competences are associated with many important areas of life, such as school readiness, academic success or sociability. Numerous intervention programs aiming to improve these capacities have emerged and have been shown to be effective. As inhibition in particular, is closely related with social cognition competences, we developed a training program that targets both abilities and implemented it in kindergarten and lower primary school classes for 6 months. We evaluated its effectiveness at improving inhibition and social cognition as well as its possible impact on academic performance. The results showed that tackling inhibition and social cognition in the classroom at an early age improved inhibition, visual attention and flexibility as well as Theory of Mind and social information processing skills. However, the impact on academic learning was weak; a slight effect on a mathematical task was observed.

## Introduction

In children, executive functions (EF) allow them to control their behavior and their attention (Espy, 2004; Burgess and Simons, 2005; Riggs et al., 2006b). For example, thinking before acting rather than acting impulsively, delaying the arrival of a reward (being able to wait longer for a bigger reward instead of getting a smaller reward directly) and/or resist temptations (being able to wait for the parents’ authorization during the aperitif to start eating). EFs also allows children to focus and maintain their attention on a task while resisting distractions during school lessons. This allows them to stay focused on what the teacher says or on the task at hand, but also remember and follow instructions correctly. That way they are able to take turns, not reacting impulsively in the playground, etc. EFs play a role not only at the level of ability to concentrate, but also at the behavioral level: they act as a true regulator of behavior (Anderson, 2002). Typically, three main components of EFs are distinguished: working memory, inhibitory control, and cognitive flexibility (Miyake et al., 2000; Diamond, 2013). Yet, according to Diamond (2013) the two basic EF are inhibition and WM while flexibility would build on these two EF and develop much later (Davidson et al., 2006).

These abilities are associated with many areas of life, e.g., school readiness (Blair, 2002) academic success (Miyake et al., 2000; Duckworth and Seligman, 2005; Duncan et al., 2007; Loosli et al., 2012) achievement, health, wealth (Moffitt et al., 2011) sociability (Hughes and Dunn, 1998), and behavior (Anderson, 2002; Pauli-Pott and Becker, 2011). As it seems important for children to be equipped with strong EF, several programs have been developed to improve it, each of them being centered on some specific EF, such as working memory (Klingberg et al., 2005; Thorell et al., 2009) attention (Rueda et al., 2005; Tamm et al., 2013) or inhibition/control capacities (Diamond et al., 2007; Tominey and McClelland, 2011; Röthlisberger et al., 2012; Volckaert and Noël, 2015, 2016). These experimental studies have shown that EF can be improved Diamond and Ling (2016) through training. In this study, we decided to focus our intervention mainly on inhibition. Indeed, although working memory plays an important role in learning and many programs have been developed to enhance these capacities, results of these interventions are quite disappointing. For instance, Melby-Lervåg and Hulme (2013) concluded their meta-analytic review of this topic by saying that memory training programs have only short-term and specific effects that do not generalize. Otherwise, Honoré and Noël tested both the well-known Cogmed program (Honoré and Noël, 2017) and another program of their own (Honoré and Noël, 2017) to enhance preschoolers working memory capacities. They found some small signs of memory improvements and barely any impact on arithmetic skills. More importantly, inhibition is a key dimension that underlies the other executive functions. In particular, inhibition is in the service of WM both by controlling goal-irrelevant information and preventing them to enter in WM and by suppressing any information in memory that is no more relevant (Halperin et al., 1999). Inhibition is also in service of flexibility as for switching from one task or one dimension to another this requires to inhibit the tendency to stay on the former task or process the former dimension.

Moreover, learning would involve both the acquisition of new knowledge of increasing complexity and the ability to resist to the interference created by the previous knowledge. For instance, in the numerical domain, the pupil first learn natural numbers and the fact that 5 is smaller than 7. Later on, the student learns rational numbers. Then, when comparing fractions for instance, such as 1/5 and 1/7, he/she has to inhibit the previous knowledge on natural numbers to correctly judge that 1/5 is larger than 1/7 (Stafylidou and Vosniadou, 2004).

This is been evidenced for instance, in reading (Ahr et al., 2016) or in number conservation or class-inclusion tasks (Borst et al., 2012). More globally, young children’s inhibitory control capacities are significantly related to their academic skills, as shown in the meta-analysis of Allan et al. (2014). Finally, in previous research, we have developed a program to enhance inhibition in young children and have observed significant improvement both on inhibition capacities but also on WM and attention (Volckaert and Noël, 2015) as well as children’ behavior.

Diamond et al. (2007) have revealed that stimulating EF within the school, and more specifically inhibition, leads children to greater academic success. Indeed, on the one hand at preschool, EFs are malleable which favors their stimulation (Sasser et al., 2017). On the other hand integrate EF program at school allows for much more intensive stimulation than when it is a program including only a few sessions.

Several researches have shown that inhibition is not independent from socio-emotional competences (SEC) (Carlson et al., 2004; Henning et al., 2010). SEC include emotional regulation, social adaptation and social cognition skills. Social cognition refers to two major models, Theory of Mind (Premack and Woodruff, 1978; Flavell, 1999) and Social Information Processing (Crick and Dodge, 1994). The first of these focuses on the child’s knowledge about mental states such as desires, beliefs, perceptions, knowledge, thoughts, intentions and emotions, and on his/her capacity to attribute mental states to others and to link them to their behaviors. The second describes the mental process involved when a child faces critical social situations and distinguishes five successive steps (Yeates et al., 2007): encoding process (encoding of relevant information), representation process (interpretation of the information), response search process (choice of desired goal), response decision process (choice of a response in line with the goal) and enactment process (behavioral response). Having good ToM (Crick and Dodge, 1994; Deneault et al., 2011; Deneault and Ricard, 2013) and SIP (Yeates et al., 2007; Nader-Grosbois, 2011) skills contributes to good social adaptation. Social cognition training programs have also been developed and proved to be effective at enhancing ToM and SIP competences. For example, after a ToM training, children’s level of socio-emotional competences increased (Izard et al., 2008; Webster-Stratton et al., 2011). An improvement in social problem solving was also found after a training program in which children discussed stories about peer interactions and performed related activities or after children had been involved in role playing (Bhavnagri and Samuels, 1996; Webster-Stratton et al., 2011). Recently, Houssa and Nader-Grosbois (2016) developed a training combining a ToM training with a SIP training and showed that receiving this combined training let to better ToM abilities, more appropriate emotion regulation, and improved social adjustment and competences in children (Houssa and Nader-Grosbois, 2016).

The intervention studies mentioned here above considered either the EF (in general or not) or social cognition. However, several researches have shown that these cognitive dimensions are not independent from one another. Significant correlations between measures of these two dimensions have been reported (Carlson et al., 2004; Benson et al., 2013; Van Nieuwenhuijzen et al., 2015). For instance, children’s executive functioning correlates with their ability to control disruptive behavior (Cole et al., 1993) and having weak EF is associated with lower emotional regulation abilities (Cole et al., 1993). Indeed, taking another person’s perspective for instance requires inhibitory control (Aïte et al., 2016). Moreover, SEC and EF processes activate the same brain region, the frontal lobe (Welsh et al., 1991). It has also been shown that the executive capacities of young children (5–7 years old) predict behavior and social competences 2 years later (Nigg et al., 1999). In the same way that an EF training would benefit from including an aspect of SEC, a training program tackling SEC would benefit from including EF (Riggs et al., 2006a). These authors suggest that EF could act as a moderator (pre-existing EF impact the relationship between intervention and social cognition outcomes), a mediator (EF interfere with the way the intervention influences the outcomes) or an outcome (social cognition intervention impacts EF) in SEC intervention studies. Therefore, it seems important to address social and emotional issues in an EF training program.

Furthermore, Kloo and Perner (2003) investigated the transfer of training between ToM and EF and found a reciprocal effect after a short training (one session). These authors explained this interdependence between the two processes by the fact that understanding the mind presupposes a certain level of executive control, and inversely executive control presupposes a certain level of insight into the mind. For instance, develop ToM competencies requires the inhibition of his/her own perspective in order to be able to take the other’s perspective. Moreover, manage his impulsivity could help in social problem solving. Houssa et al. (2017) compared one program targeting inhibition (Volckaert and Noël, 2015) and one focusing on social cognition (Houssa and Nader-Grosbois, 2016). They showed that while the social cognition program increased social cognition skills, the inhibition intervention not only improved executive abilities such as inhibition, attention or working memory but also led to enhanced emotional regulation and social adaptation. As pointed out by Diamond and Ling (2016) to be effective, an intervention program aiming at improving EF should not only train EF abilities but also promote factors supporting them. For instance, stress (Arnsten, 1998) negative mood (Gable and Harmon-Jones, 2008; Desseilles et al., 2009) and lack of social support (Cacioppo and Patrick, 2008) negatively affect executive abilities.

Both EF and ToM seem to predict academic success: they are related to mathematics and literacy (Bull and Scerif, 2001; Gumora and Arsenio, 2002; St Clair-Thompson and Gathercole, 2006; Blair and Razza, 2007; Protopapas et al., 2007; Blair and Diamond, 2008; Monette et al., 2011; Shaul and Schwartz, 2014). To take the example of SEC, anxiety impacts performance in academic tests (McDonald, 2001) and the association of negative affect with academic tasks (e.g., class participation, quizzes or tests) interferes with performance in language and mathematics (Gumora and Arsenio, 2002). Researchers have shown that emotion regulation skills are related to academic success because they impact the relationship between teachers and pupils (Graziano et al., 2007), cognitive processes (such as attention, working memory, inhibition) (Graziano et al., 2007; Denervaud et al., 2017) and engagement with learning (Graziano et al., 2007; Pekrun and Linnenbrink-Garcia, 2014) all of which are necessary for school learning. Although emotions can sometimes interfere with learning, experimental results also suggest that they often facilitate cognitive processes, such as attention and memory, which are essential for learning (Denervaud et al., 2017).

The aforementioned training studies (Röthlisberger et al., 2012; Healey, 2013; Houssa and Nader-Grosbois, 2015, 2016; Volckaert and Noël, 2015, 2016) have examined the impact of EF or ToM and/or SIP training implemented in small groups outside the classroom. However, implementing an intervention program at school leads to more stimulation and, more importantly, to a greater possibility of transfer to academic learning. Some authors have developed school curriculum programs that aim to improve EF or SEC and observed a positive impact on these trained competences (EF: Diamond et al., 2007; Barnett et al., 2008 and social problem solving: Domitrovich et al., 2007; Merrell et al., 2008; Webster-Stratton et al., 2008; Theurel and Gentaz, 2015). Moreover, Riggs et al. (2006b) showed that stimulating children’s SEC in class enhances inhibition abilities.

Finally, in their review, Diamond and Ling (2016) have highlighted the importance of the amount of practice time required for an EF training program to be effective; the longest training programs lead to better EF outcomes. The training program developed for this study was therefore implemented once a week in the classroom for 6 months, for a total of 18 sessions.

The aim of the present study was to assess the effectiveness of an intervention program combining the stimulation of inhibition and social cognition (ToM and SIP). As inhibition (Dowsett and Livesey, 2000; Carlson, 2005) and social cognition (Wellman, 1991; Lemerise and Arsenio, 2000; Baron-Cohen, 2001) show significant growth during the preschool period, and are hence malleable, the present intervention took place with young children (5 and 6 years old). Literature has shown how difficult it is to highlight significant results on working memory in young children (Melby-Lervåg and Hulme, 2013). We then choose to focus on inhibition because many research have found that targeting the inhibition dimension of the EF is very promising (Diamond et al., 2007; Volckaert and Noël, 2015) and also because this study combines the content of the studies of Volckaert and Noël (2015) and Houssa and Nader-Grosbois (2016) which specifically targeted inhibition for the EF part. We also know the importance of developing good inhibition capacities to have good social adaptation abilities. Flexibility was, however, tested because when certain training games became more complex, the rules then implied flexibility. In terms of attention, we have chosen to look at the impact of training on these skills because research has shown that stimulating inhibition has a significant impact on attention capacities (Noël et al., 2007).

In Volckaert and Noël (2015) and Houssa and Nader-Grosbois (2016) 5- and 6-year-old children were then assigned either to an experimental condition in which they received the intervention program once a week for 6 months, or to a control condition in which they engaged in normal activities in class. Just before and after the intervention, the children in the experimental and control groups were tested for their performance in EF and social cognition. Then, as EF and social cognition both have important implications for school success (Gumora and Arsenio, 2002; Blair and Diamond, 2008) participants were also tested for their academic performance (literacy and mathematics). Positive effects of the program were expected in the trained competences, as it has been shown that EF and social cognition skills can be trained (Röthlisberger et al., 2012; Houssa et al., 2013; Tamm et al., 2013; Houssa and Nader-Grosbois, 2015; Volckaert and Noël, 2015, 2016). A transfer effect was also predicted in tasks assessing literacy and mathematics, as previous researchers have shown that EF and social cognition are related to academic performance (Bull and Scerif, 2001; Gumora and Arsenio, 2002; St Clair-Thompson and Gathercole, 2006; Blair and Razza, 2007; Graziano et al., 2007; Protopapas et al., 2007; Monette et al., 2011; Shaul and Schwartz, 2014; Denervaud et al., 2017).

## Materials and Methods

### Participants

As the study took place in classrooms, the participants were recruited through their school. Schools were contacted by email or by phone. They were presented with the project and to take part in it. There were four inclusion criteria for schools: they must be part of the French speaking part of Belgium, they must provide both kindergarten and the first year of elementary schooling, there must be at least two classes in each grade, and they must use conventional teaching (no alternative pedagogy). Furthermore, we paid attention to vary socio-economic status of schools and regions. If the school’s director was interested in the project, the intervention was presented to concerned teachers.

Exclusion criterion by children was language disorders or intellectual disabilities. Children were excluded if they have not elementary comprehension and production of spoken French.

Among the schools meeting these criteria, nine were randomly selected. All parents of children in the last (third) year of kindergarten (K3) and in the first year of primary school (P1) were given an information letter presenting the research as well as a consent form. Among the children whose parents gave their written informed consent, a maximum of eight children per class were randomly selected to participate in the study. Data were collected from 241 preschoolers (51% boys) aged between 4 years and 9 months and 7 years and 6 months old (*M* age = 69.08 months, *SD* = 7.32 months). Hundred and twenty children were in K3 and 121 children in P1. Parents’ level of education was evaluated on a scale (from elementary school not completed to university degree). In average, mothers indicated 4.74 (*SD* = 1.24) (5 corresponded to “3 years of Graduate school”) and fathers indicated 4.70 (*SD* = 1.28). Concerning the family’s monthly income, parents had to specify it on a scale from “0 – 999€” to “6000€ or more,” with a mean on 4.11 (*SD* = 1.64) (4 corresponding to 3000 – 3999€ a month), which is a little bit above the average of the country. Children were all Caucasians.

The study was approved by the ethics committee of the Psychological Sciences Research Institute of UCLouvain.

### Procedure

The study took place in the schools. In each grade of each school, one class was assigned to the experimental group (121 children), which received the intervention program, and one class to the control group (120 children), which engaged in usual classroom activities.

The research consisted of three phases: pre-test, intervention and post-test. For the pre-test session, different tests were initially administered individually across two sessions for each participant (lasting approximately 30–40 min according to the participant’s attention and availability).

The children were pre-tested during the first 5 weeks (September – October), the intervention program took place during the next 6 months for the experimental group (November – April), and the children were then post-tested during the last 4 weeks (May). The program consisted of eighteen 50-min sessions implemented in the classroom in the presence of the teacher.

Within each class, sessions were administered by the same experimenter (three Ph.D. in Psychology). Pre- and post-test were performed blind.

#### Pre- and Post-testing

##### Instruments for the inclusion criterion

The validated scales of the Wechsler Intelligence Scales – third edition (Weschler, 2004) were used in a pre-test session to exclude possible mental retardation. We used two subscales which were “Information” from the verbal scale and “Block design” from the performance scale. Standard scores for these subtests have a mean of 10 ± 3. A global score was calculated as the mean of the two standard scores. To take part in the study, children had to be in the normal range (±1.5 SD), i.e., to have a global score lying between 5.5 and 14.5. In terms of validation, the intercorrelations calculated between raw scores on all scales were high.

#### Executive Functions

##### Attention

Executive attention is often considered among the EF (see for instance Diamond, 2013) as it requires the inhibition of external distractors to filter the information to be processed; this is typically called selective attention. Visual attentional capacities were then assessed with the Face Cancelation Task (Brooks et al., 2009). In this task, participants were presented with an A3 sheet displaying 96 similar faces organized into 8 lines of 12 faces. Two target faces were presented at the top of the sheet and participants were asked to cancel, any of the 96 faces which were identical to either of the target ones, in a maximum of 180 s. They were asked to do this as quickly and accurately as possible. Accuracy [= correct responses (maximum 20) – errors] and the time taken to perform the task were calculated and an efficiency score (ES = accuracy/time) was used as the dependent variable. The Face Cancelation Task is one of the 32 subtests of the NEPSY-II, validated by 1200 children between 3 and 16 years old. The test–retest reliability and internal validity were high.

##### Inhibition

Three tasks were used. First, a Stroop Task developed by Catale et al. (2014) was chosen as it did not require the participants to read. The validation was performed with TD children and ADHD children (Catale et al., 2014). The task was composed of three parts. In the color denomination part, participants had to name the color of 45 colored rectangles (red, yellow, and green). In the black fruit part, they had to name the real color of 45 fruits colored black (strawberry, banana, and pear). In the interference part, they had to name the real color of 45 fruits displayed in an incorrect color (yellow strawberries, green bananas, and red pears). In each part, participants were asked to perform as quickly and accurately as possible. Time as well as accuracy [45 – corrected (0.5) and uncorrected errors (1)] were scored. An ES was computed (accuracy/time) for the interference part and used as the dependent variable. Second, in the Tongue Task (Willoughby et al., 2011) participants were told “we are going to play a game together in which we will have to keep a piece of candy on our tongue as long as possible, without chewing it, sucking it or swallowing it.” In a first 10-s trial, both the experimenter and the child placed a sweet on their tongue, keeping their mouth open. If the child closed his/her mouth for more than 3 s, the experimenter reminded him/her to open it. If the child kept the sweet on his/her tongue for 10 s, the experimenter told him/her that he/she had won and could eat it. Then, a 40-s test was administered and the child was reminded of the rules: *“You have to hold with the sweet on your tongue, with your mouth open, for as long as you can until I tell you can stop, without closing your mouth, and without sucking, chewing or swallowing the sweet.”* The time in seconds during which the child followed the instructions was used as the dependent variable. As performance were at ceiling at pre-test (more than half of the participants held the sweet for 40 s), the duration of the task was lengthened to 60 s at post-test. These analyses therefore were applied only on performances displayed by the participants who did not reach 40 s at pre-test. Tongue Task was validated by 926 3–6 years old children through factorial analysis and convergent validity. Third, the Teddy Delay Task (Sonuga-Barke et al., 2003) was a computerized task assessing delay of gratification by asking participants to make a choice between a small immediate reward (one reward after 1 s) and large delayed rewards (2 rewards after 17 s). This choice was represented by two teddy bears, one holding one balloon in the foreground of the left side of a screen (small immediate reward) and one holding two balloons in the background of the right side of a screen (large delayed reward). When the participant made his/her choice, the experimenter clicked on the chosen bear, which started walking and released its balloon(s). The participant received one token for each balloon released and was told that he/she could exchange these tokens for stickers at the end of the game. Two practice trials were first presented; in the first, the child was invited to choose between the two bears, while in the second, he/she had to choose the one not previously chosen in order to experience both waiting times. There were 20 test trials and the percentage of choices of the large delayed reward was computed. The test–retest reliability is satisfactory (*r* = 0.67) (Dalen, 2004).

##### Flexibility

Two tasks were used. First, in the Dimensional Change Card Sort (Zelazo, 2006) participants were presented with two target cards, one with a blue rabbit and one with a red boat, and two sorting trays. In the first part, the color game, the child had to sort eight testing cards (four red rabbits and four blue boats) according to their color: if the card was blue, the child had to put it in the sorting tray below the blue rabbit and if it was red, he/she had to put it in the sorting tray below the red boat. The first two cards (one blue and one red) were used as practice trials. In a second part, the shape game, the child had to sort six cards according to their shape: if the card depicted a rabbit, he/she had to put it in the sorting tray below the blue rabbit and if it showed a boat, he/she had to put it in the sorting tray below the red boat. Then, a third part was presented to the child if he/she had successfully completed the second one (at least 5 correct responses out of 6). In this part, 12 cards were successively presented; half of them had a border and the other half did not. When the card had a border, the child had to play the color game and when it did not have a border, he/she had to play the shape game (Zelazo, 2006 for more details). The score of the third part (maximum = 12) was recorded to assess flexibility. Doebel and Zelazo (2015) validated the DCCS and revealed a perfect inter-rater reliability.

Second, the Traffic Lights Task (Volckaert and Noël, 2015) was a computerized task inspired by the Dots Task (Davidson et al., 2006) composed of three conditions. In the congruent condition, a green traffic light appeared either on the right or left side of the screen and the child had to press a button on the side of the traffic light as fast as possible. In the incongruent condition, the traffic light was red and the child has to press a button situated on the opposite side from the traffic light as fast as possible. In the mixed condition, there were both green and red traffic lights and the child had to press a button on the side of the traffic light when it was green and on the opposite side from the traffic light when it was red. Reaction times and number of correct responses were recorded and an ES was computed (correct responses/median reaction time) and used as the dependent variable.

#### Social Cognition

##### Theory of mind

We used the translated version (Nader-Grosbois and Houssa, 2016) of the ToM Task Battery (Hutchins et al., 2008) as a direct measure of the children’s understanding of Theory of Mind. This consisted of 15 questions addressing different mental states and with increasing difficulty, from facial emotion recognition to inference of second-rate false beliefs. The total score (maximum = 15) was used as the dependent variable. This test was validated through test-retest reliability, internal consistency and external correlation.

##### Social information processing

The Social Problem Resolution Task (Barisnikov et al., unpublished) assesses the ability to judge the appropriateness of the social behavior of others and determines the extent to which the judgment is based on knowledge of conventional and/or moral rules. It consists of 14 illustrations of everyday social situations in which the character displays a behavior which is either appropriate (5 situations, for example of sharing or of help between two protagonists) or inappropriate (9 situations, for example, frustration, conflict or non-respect of social rules). For each fictious situation, the children were asked three questions. First: *“Can you see what is happening in this image? Is he/she behaving well or not?”* This question aimed to explain what happens in a given situation. A correct response was scored 2 and an incorrect response was scored 0 (for all situations, identification score maximum = 28). Second: *“Can you show me what is appropriate/inappropriate?”* This question measured the capacity to qualify the target behavior in one protagonist, as socially appropriate or inappropriate behavior, toward the other protagonist. It was scored 1 (correct response) or 0 (incorrect response) (for all situations, judgment score maximum = 14). Third: *“Why is it appropriate/inappropriate?”* This third question assessed the level of complexity of the child’s judgment and in which measure he or she was able to refer to social/moral rules in his or her justification about the protagonist’ behavior. This question was scored 2, 5, or 7, according to the level revealed by the child’s answer (for all situations, justification score maximum = 98). The descriptive level (maximum 2 points) corresponded to a description by the child about what he/she saw in the illustrated situation. At the intersubjective level (maximum 5 points), the child gave an explanation showing his or her attention on relational and social aspects between the protagonists, and his or her to social consciousness. At the conceptual level (maximum 7 points), both social consciousness and reference to social or moral rules underlay the child’s explanation. If the child could not answer or if his/her answer revealed a misunderstanding of the situation, no point was given. A total score (maximum = 10) was calculated for each situation and the mean score, computed for appropriate behaviors and for inappropriate behaviors, was used as the dependent variable. The validation was performed with TD children and people with intellectual disability. The inter-judge agreement was 98% congruent (Hippolyte et al., 2010).

#### Academic Learning

##### Language

For participants in K3, we used the Image Designation Task from the ELO (Khomsi, 2001) in which words were verbally presented to children and they had to point to one of four images that corresponded to the given word. The task consisted of 20 items; 1 point was given for every correct answer, and the percentage of correct responses (%CR) was computed. In P1, children were presented with the Reading Task from the BELO (Pech-Georgel and George, 2006) divided into two parts: letter (26 items) and word (12 items) reading. Each correct response corresponded to 1 point (maximum = 38) and the % CR was calculated. The BELO was validated through a good external validity and a good internal consistency (Pech-Georgel and George, 2006).

##### Mathematics

K3 participants were evaluated for their performance in a number conservation task and a simple addition task. We used the Number Conservation Task from the TEDI-MATH (Van Nieuwenhoven et al., 2001). The validation was performed on a large sample of TD children and showed good internal consistency (Van Nieuwenhoven et al., 2001). In the initial setting, two parallel rows of 6 tokens each (equally spaced) were arranged between the child and the experimenter and the child was asked whether the two rows were numerically equivalent or whether one was larger or smaller than the other. Then, in a first condition, the experimenter spaced the tokens of one of the rows and asked again the child if they were numerically equivalent or if one was larger than the other. Then, tokens were rearranged as in the initial setting and the same question was again asked to the child. Finally, in the second condition, one of the rows was put into a pile and the child was again asked the same question. For the two conditions, 0 points were given if the child said the collections were not equivalent in number; 1 point was given if the child said they were numerically equivalent but needed to count them first and 2 points were given if the child said they were numerically equivalent and gave a logical justification (e.g., *“you did not add or remove any token”*); the total score was computed (max = 4). In the Simple Addition Task, developed by Noël (2009) children were presented with 10 additions (4 ties: 2 + 2, 3 + 3, 4 + 4, and 5 + 5, and six non-ties: 2 + 3, 2 + 4, 2 + 5, 3 + 4, 3 + 5, 4 + 5). For each addition, the child was presented with a drawing of apples corresponding to the first operand; the experimenter said: *“Here are* [number of apples] *apples; if I give you* [second operand] *more apples, how many apples will you have in total?”* The child’s answers and strategies used to solve the problem [(1) counting all, e.g., for 2 + 3 “1,2,3,4,5”; (2) counting on = counts from the first addend, e.g., “2,3,4,5”; (3) counting min = counts from the larger addend, e.g., “3,4,5”; (4) mental strategy] were recorded. A score combining the accuracy of the answer and the strategy used to solve the problem (0 = wrong answer; 1 = correct answer using strategy 1; 2 = correct answer using strategy 2; 3 = correct answer using strategy 3; 4 = correct answer using strategy 4) was computed and used as the dependent variable.

Participants in P1 were presented with an Arithmetic Problems Task divided into two parts: additions and subtractions. The addition task consists of 15 problems presented in order of increasing difficulty; the first group involved operands less than 5 (2 + 2, 3 + 3, 4 + 4, 2 + 3, 2 + 4, 3 + 4); in the second group, one operand was less and the other one was greater than 5 but the sum was less than 10 (2 + 5, 3 + 5, 4 + 5); and in the third group both operands were greater than 5 (6 + 6, 7 + 7, 8 + 8, 6 + 8, 7 + 8, 8 + 9). For the first and third groups of items, problems using both ties and non-ties used, the former being presented first as they are easier. Subtractions correspond to the counterpart of the additions (e.g., 8-3 as the counterpart of 3 + 5). A stop criterion was applied after three consecutive failures. The total number of correct responses was calculated (max = 30).

#### Intervention

The intervention program consisted of eighteen 45-min sessions implemented in the classroom in the presence of the teacher. Once a week during 6 months, a trained psychologist presented each session with fun activities aiming at improving inhibition and social cognition skills. In each session, both inhibition and social cognition were trained; the inhibition aspect of the program was inspired by Volckaert and Noël (2015, 2016) training program and the social cognition aspect by Houssa et al. (2013) and Houssa and Nader-Grosbois (2015). As difficulty should progressively increase and correspond to the zone of proximal development (Vygotsky, 1986) activities were presented in order of increasing difficulty, according to the theory underlying each concept. We choose to target inhibition in the activities, as this is described as one of the core EF (Diamond, 2013) and is particularly related to SEC (Riggs et al., 2006a). The four components of inhibition were trained: inhibition of a predominant response, interruption of an ongoing response, inhibition of external distractors and impulsivity control. The executive functions drill was accompanied by metacognition, with the children being encouraged to analyze the mental processes happening when carrying out an activity. Flavell (1979) already reported that young children have limited knowledge of their own functioning, therefore weak metacognitive capacities. However, metacognitive skills are essential from an early age, both at home and school, in non-routine executive tasks or even during new learning. Understanding his own functioning allows not only the child to manage a task but also to promote the transfer of acquisitions when the child understands that it could possibly be applied to other tasks. The transferability could help the child to better self-regulate in problem solving, in new learnings and perhaps, his or her emotions in new situations. Metacognitive abilities and transferability could contribute to academic success in middle and long-term. For example, the use of metacognition in training has already shown its benefits on mathematical reasoning for example (Kramarski and Mevarech, 2003; Teong, 2003). The use of metacognition as we used it in our intervention makes it possible both to bring the child to understand the mechanisms involved in each proposed exercise but also to keep each child in the “active” mode as you will see. Three characters, inspired by Reflecto (Gagné and Longpré, 2004) symbolizing different aspects of inhibition were used. First, Mr. Stop represented the ability to inhibit a predominant response; he invited the children to take time before acting: “Stop: first I think and then I do.” The Detective referred to the capacity to inhibit interference and distractors in order to focus attention on elements relevant to the ongoing task and encouraged the children to check his work. Finally, the Statue corresponded to motor control; it invited the children not to move excessively during calm activities, and to observe which parts of their body were in movement.

Before each activity focusing on the EF or the social cognition abilities, children were reminded to activate and use the characters useful for the task.

The social cognition program was developed with reference to a theoretical background for both ToM and SIP competences. The activities involving ToM competences were based on Howlin et al. (2017) program which suggests a progression in terms of theory of mind on emotions and their links with other mental states: (1) recognition of emotions expressed by faces on photos, (2) recognition of emotions expressed by “schematic” faces, (3) understanding of causes and consequences of emotions in social situations, (4) understanding of a desire based on emotions and (5) understanding of a belief based on emotions and beliefs (1) simple perspective taking, (2) complex perspective taking, (3) seeing leads to knowing, (4) true belief/action prediction, and (5) false belief (Hadwin et al., 1996). The exercises targeting the SIP competences were built according to the six steps of the SIP model (Crick and Dodge, 1994): (1) encoding other people’s social cues, (2) interpretation of social cues, (3) clarification of goals, (4) response access, (5) response decision, (6) behavioral execution. Finally, the proposed activities were presented in order of increasing difficulty, in accordance with the hierarchical levels of justification distinguished by Barisnikov et al. (unpublished) in the RES: (1) descriptive level, (2) intersubjective level, and (3) conceptual level. The activities tackling ToM and SIP competences included pictures, sequences of play, video extracts, handling of objects, story reading and other activities.

The program was used in the classroom; working in groups allowed socio-cognitive conflicts to emerge which might help children to become aware of the diversity of points of view in a given situation. Socio-cognitive conflicts is interesting because this generally conducts to a positive influence of social interactions on learning. Moreover, authors of a recent meta-analysis on training in understanding emotions explain that the effect sizes are larger when children are trained in groups (Sprung et al., 2015) rather than individually.

Teachers were invited to use the concepts and materials used in the session as often as possible during the rest of the week to promote a transfer of knowledge to other situations. To help teachers ensure this continuity, activities were suggested every week (e.g., story reading followed by a discussion, use of metacognition characters in different situations; finishing an activity started in the session).

Teachers were asked to keep a record book each week. They were invited to describe their general feeling about the activities and children’s receptiveness, to write down any comments or questions and to specify what concept/material was used in the classroom during the week (by ticking a box corresponding to the given concept/material). Indeed, the teacher was expected to continue the activities during the week, targeting the concepts learned during the session. This should then be reported in the logbook.

Finally, the level of involvement in the project of teachers in the experimental classes was assessed. The measure, ranging from 1 to 5 (Table 1), considered the teachers’ involvement during the sessions (Is s/he present? Does s/he take an interest in the session? Does s/he participate?) and outside the sessions (use of the tools and concepts during the week). The assessment on this scale was based on the observation of the teacher during the session and on the contents of the record book for involvement outside the sessions.

**TABLE 1:** Measure of the involvement of the teachers in the project.

**Level**	**Description**
1	Minimum involvement during the sessions, no (or little) use of the materials and concepts outside the sessions.
2	Weak involvement during the sessions, little use of the materials and concepts outside the sessions.
3	Average involvement during the sessions, little or occasional use of the materials and concepts outside the sessions.
4	Intense involvement during the sessions, little or occasional use of the materials and concepts outside the sessions.
5	Intense involvement during the sessions, intense use of the materials and concepts outside the sessions.

Finally, the attendance of the participants of the study was recorded at each session.

## Results

First, we were interested in the implementation of the intervention program. We checked whether the participants in the experimental group received a sufficient number of sessions by exploring the variable “attendance” and whether the teachers were involved in the project through the variable “level of involvement of the teachers.”

Second, a first series of *t*-tests and Chi squares were calculated to compare the characteristics of the experimental and control groups in terms of demographic data (age, sex, and school year) and pre-test performance. The effect of the parents’ level of education and family incomes were evaluated but there was no significant influence of those variables (Table 10).

Third, to assess the effect of training, we conducted repeated-measures ANOVAs with testing time (pre-test and post-test) as within-subject factors and group (experimental and control) as between-subject factor on each of the baseline task scores; partial Eta-squared was calculated as a measure of effect size. For the RES, two within-subject factors were introduced: testing time and appropriateness of behavior (appropriate and not appropriate).

Finally, to better understand which children benefited the most from the intervention, we evaluated the potential impact of characteristics concerning the teachers (involvement of the teacher in the project and number of years of service) and the children (initial level of EF and of social cognition competences, age, school year, attendance at sessions) on the progression in EF and social cognition skills.

The normality assumption was controlled for each variable.

### Implementation of the Program

#### Attendance

Most of the children in the experimental group attended most of the program. As shown in Table 2, 85% of participants attended 16 or more of the 18 sessions.

**TABLE 2:** Number of sessions that children from the experimental group attended.

**Number of sessions**	**Number of children**
12	2
14	7
15	9
16	14
17	40
18	48

#### Level of Involvement of the Teachers

The mean level of involvement (3.31 ± 1.40) corresponds to “average involvement of the teacher during the sessions and occasional use of materials and concepts outside the sessions.” As shown in Table 3, 77% of children had teachers with at least this level of involvement.

**TABLE 3:** Level of involvement in the project of teachers of children in the experimental group.

**Level of involvement**	**Number of teachers**	**Number of children**
1	3	22
2	1	8
3	5	33
4	4	29
5	4	30

### Control and Experimental Groups Comparisons at Pre-test

The two groups were statistically equivalent in terms of demographic data, verbal and non-verbal intelligence (see Table 4) and pre-test performance (Table 5) on the executive, socioemotional and academic tasks.

**TABLE 4:** Demographic data at pre-test (means ± standard deviation) and comparison of the two groups (*t*-test or Chi^2^ and *p*-value).

**Demographic data**	**Experimental group**	**Control group**	***t*/Chi^2^**	***p***
Age (in months)	69.02 ± 7.20	69.08 ± 7.48	−0.06	0.951
Sex	63 girls – 58 boys	55 girls – 65 boys	0.94	0.368
School year	63 M3 – 58 P1	59 M3 – 61 P1	0.04	0.898
WPPSI verbal	9.02 ± 2.37	9.17 ± 2.58	−0.47	0.679
WPPSI visuo-spatial	8.98 ± 2.55	9.13 ± 2.75	−0.41	0.638

**TABLE 5:** Means ± standard deviations for each task at pre-test and post-test in the two groups and comparison of groups at pre-test (*t*-test and *p*-value).

**Measures and tasks**	**Experimental Group**			**Control Group**			**Comparison at pre-test**	
	***N***	**Pre-test**	**Post-test**	***N***	**Pre-test**	**Post-test**	***t***	***p***
**EF**								
Face cancelation task	121	0.033 ± 0.048	0.072 ± 0.045	119	0.039 ± 0.041	0.065 ± 0.046	−1.07	0.284
Fruits Stroop	117	0.45 ± 0.13	0.56 ± 0.14	114	0.45 ± 0.14	0.56 ± 0.16	−0.01	0.999
Tongue task	30	23.13 ± 11.69	59.83 ± 0.91	17	26.18 ± 12.64	50.18 ± 15.86	−0.83	0.409
Teddy	121	45.12 ± 15.67	40.12 ± 16.30	120	45.33 ± 13.78	39.71 ± 18.72	−0.11	0.912
DCCS	108	6.29 ± 1.66	7.77 ± 2.44	100	6.50 ± 2.11	7.20 ± 2.15	−0.81	0.422
Traffic lights	120	0.025 ± 0.008	0.030 ± 0.009	118	0.024 ± 0.007	0.028 ± 0.009	1.46	0.236
**Social cognition**								
ToM Battery	121	8.98 ± 2.43	11.07 ± 2.27	120	9.14 ± 2.46	10.66 ± 2.14	−0.50	0.616
RES	121	4.95 ± 0.86	5.72 ± 0.86	119	4.76 ± 1.05	5.27 ± 1.02	1.55	0.124
**Academic learning**								
Language K3	61	73.20 ± 10.49	78.85 ± 9.72	58	74.66 ± 10.25	79.48 ± 8.36	−0.77	0.554
Language P1	60	33.07 ± 19.07	78.11 ± 15.71	59	32.43 ± 19.81	75.29 ± 16.10	0.18	0.857
Number conservation K3	61	0.62 ± 1.10	1.31 ± 1.71	58	0.40 ± 0.88	0.79 ± 1.33	1.25	0.215
Additions K3	61	0.56 ± 0.65	1.43 ± 1.08	58	0.65 ± 0.77	1.37 ± 1.11	−0.71	0.480
Arithmetic P1	60	8.25 ± 7.31	19.35 ± 5.71	60	9.32 ± 6.62	20.45 ± 5.50	−0.84	0.404

### Effects of the Intervention

Table 6 presents the results of the repeated measure ANOVAs comparing the pre- and post-tests for the control and experimental groups. As can be seen, a significant effect of time was observed in all the test tasks except the Teddy Task. For the Teddy Task, participants chose the large delayed reward significantly less at post-test compared to pre-test.

**TABLE 6:** Repeated-measures analyses for each task.

**Measures and tasks**	**Effect of time**			**Effect of group**			Effect of interaction group*time		
	***F***	***p***	**η ^2^**	***F***	***p***	**η ^2^**	***F***	***p***	**η ^2^**
**EF**									
Face cancelation task	113.30	<0.001	0.429	0.01	0.980	0	4.01	0.046	0.017
Fruits Stroop	171.89	<0.001	0.429	0.01	0.920	0	0.05	0.829	0
Tongue task	195.85	<0.001	0.813	1.79	0.188	0.038	8.57	0.005	0.160
Teddy	15.32	<0.001	0.060	0.01	0.948	0	0.05	0.818	0
DCCS	75.39	<0.001	0.242	3.17	0.076	0.013	0.30	0.583	0.001
Traffic lights	28.12	<0.001	0.128	0.69	0.409	0.003	3.87	0.051	0.018
**Social cognition**									
ToM Battery	155.91	<0.001	0.395	0.24	0.624	0.001	3.95	0.048	0.016
RES	92.17	<0.001	0.279	10.92	0.001	0.044	3.34	0.069	0.014
**Academic learning**									
Language K3	26.79	<0.001	0.186	0.50	0.480	0.004	0.17	0.683	0.001
Language P1	709.47	<0.001	0.858	0.38	0.538	0.003	0.44	0.510	0.004
Number conservation K3	10.92	0.001	0.085	4.70	0.032	0.039	0.79	0.376	0.007
Additions K3	83.42	<0.001	0.416	0.02	0.885	0	0.68	0.412	0.006
Arithmetic P1	319.19	<0.001	0.730	1.24	0.286	0.010	0.01	0.979	0

Significant effects of group were observed in the RES [with higher performance in the experimental group (5.34 ± 0.76) than in the control group (5.04 ± 0.87)] and in the Number Conservation Task [K3, with higher performance in the experimental group (0.48 ± 0.49) than in the control group 0.30 ± 0.44], and a marginal group effect was found in the Traffic Lights Task [with a slightly higher ES in the experimental group (0.028 ± 0.008) than in the control group (0.026 ± 0.007)].

**FIGURE 1:**
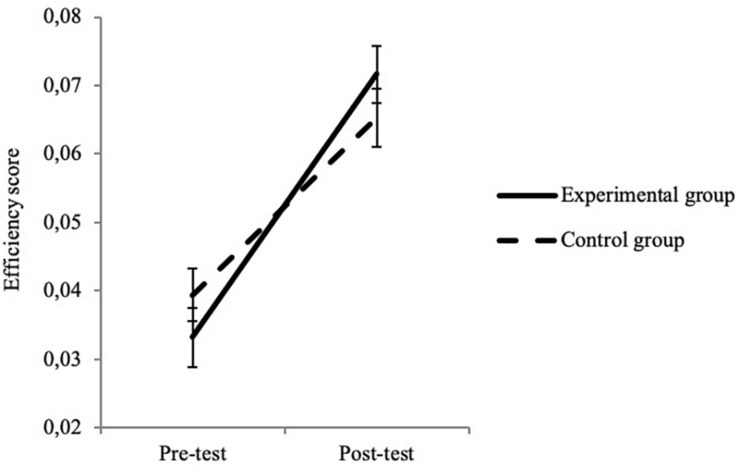
Interaction between test time and group in the Face Cancelation Task. Error bars depict standard errors.

**FIGURE 2:**
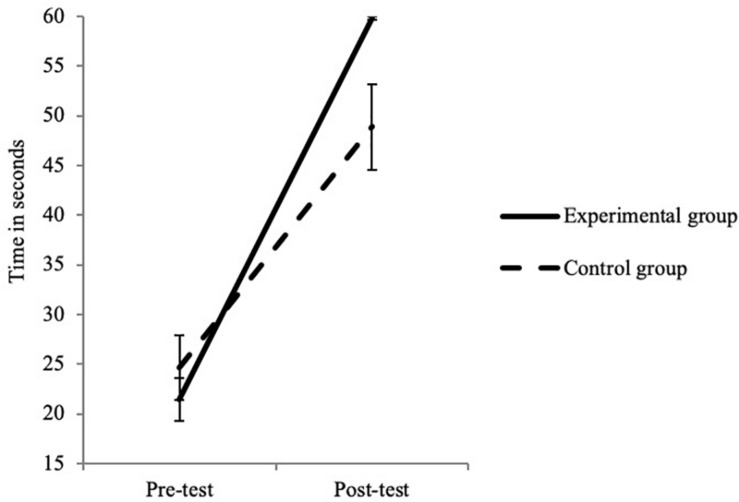
Interaction between test time and group for the Tongue Task. Error bars represent standard errors.

**FIGURE 3:**
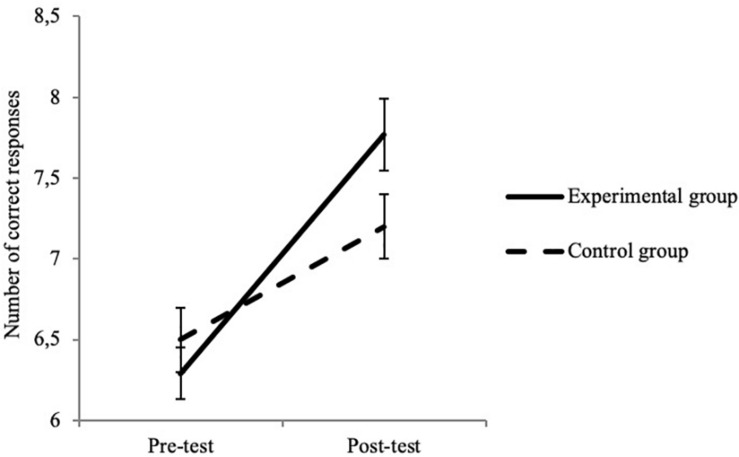
Interaction between test time and group for DCCS. Error bars represent standard errors.

More importantly, significant interactions between time and group were observed in several tasks. For the EF measures, significant interactions were found in the Face Cancelation Task, the Tongue Task and the DCCS task (Table 6). For the Face Cancelation Task (Figure 1), the improvement in ES was greater in the experimental group (0.038 ± 0.046) than in the control group (0.026 ± 0.048). In the Tongue Task (Figure 2), the duration spent with the candy in the subject’s open mouth increased more in the experimental group (38.37 ± 10.80) than in the control group (24.27 ± 19.62). Finally, in the DCCS task, we observed a greater increase of performance in the experimental (1.48 ± 2.87) than in the control (0.70 ± 2.86) group (Figure 3). These three results suggest that the intervention program was effective at improving children’s EF capacities, and in particular at improving their performance in selective attention, inhibition and flexibility.

**FIGURE 4:**
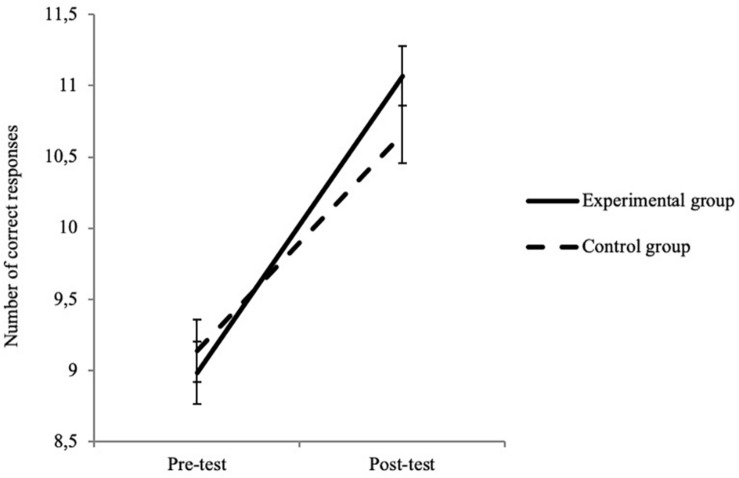
Interaction between test time and group for ToM task Battery. Error bars represent standard errors.

**FIGURE 5:**
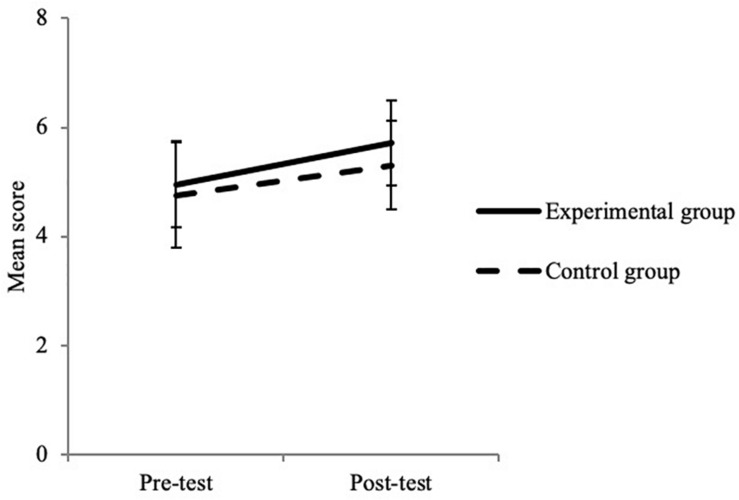
Interaction between test time and group for the RES. Error bars represent standard errors.

For social cognition, the interaction effect was significant for the ToM task battery and marginal for the RES (Table 6). These results reveal a significantly greater improvement in the experimental group (2.09 ± 2.24) than in the control group (1.52 ± 2.25) for the ToM task battery (Figure 4) and, more moderately, for the RES (Figure 5) (an improvement of 0.77 ± 0.99 for the experimental group and of 0.55 ± 1.09 for the control group).

The intervention thus successfully improved performance in ToM and in SIP.

Lastly, we looked at possible indirect effects of the intervention on academic learning. The repeated-measures analyses revealed no significant interaction effect between test time and group for any of the tasks assessing academic learning (Table 7). However, as we wanted to examine the potential effect of intervention in greater depth, and as the two groups’ performances were equivalent at pre-test, we compared their post-test performances by computing *t*-tests for independent samples. The results of these analyses (Table 7) indicate a marginal difference between the two groups in the Number Conservation Task (K3), with a higher score in the experimental group (1.31 ± 1.71) than in the control group (0.79 ± 1.33).

**TABLE 7:** *T*-tests for independent samples on academic learning tasks.

**Measures**	***t***	***p*-value**
Language K3	−0.38	0.706
Language P1	0.97	0.335
Number conservation K3	1.85	0.067
Additions K3	0.25	0.803
Mathematics P1	−1.08	0.285

These supplementary analyses suggest a slight transfer effect to numerical development.

### Impact of Teachers’ and Children’s Characteristics

An exploratory factorial analysis was computed in principal component analysis on four of the six EF tasks (Face Cancelation, Fruits Stroop, Traffic Lights and DCCS; the Tongue Task was excluded because of the small N and the Teddy Task because performances decreased from pre-test to post-test) and on the two social cognition tasks (ToM task and RES) in order to compute a single factor for performance in EF and another for performance in social cognition; these would represent the latent variable and be less sensitive to variables that were not of interest (e.g., aspects of the tasks, motivation, etc.). The factorial analyses were computed on pre-test performance; post-test factors were then calculated with the values of loadings of the pre-test factors. Change from pre-test to post-test was calculated by subtracting the pre-test factorial score from the post-test factorial score. We then computed correlational analyses between these factors and teachers’ and children’s characteristics. The sampling adequacy was calculated with the Kaiser–Meyer–Olkin which is 0.56 for the EF factor and 0.50 for the SEC factor (which is not high but not considered as unacceptable; Kaiser, 1974). The results of the factorial analyses are displayed in Table 8 for the EF factor in Table 9 for the social cognition factor.

**TABLE 8:** Task loadings for the EF factor.

**EF tasks**	**Loading on the factor**
Face cancelation task	0.329
Fruits Stroop	0.792
Traffic lights task	0.762
DCCS task	0.537
Percentage of explained variance	40.12

**TABLE 9:** Task loadings for the social cognition factor.

**Social cognition measures**	**Loading on the factor**
ToM task battery	0.828
RES	0.828
Percentage of explained variance	68.62

The correlational analyses (Table 10) showed a significant negative correlation between the change in the EF factor and the EF factor at pre-test as well as between the change in the social cognition factor and the social cognition factor at pre-test. However, all other correlations were non-significant.

**TABLE 10:** Correlations between the change in the EF factor, the change in the social cognition factor and teachers’, children’s, and family’s characteristics.

	**Change in EF factor**	**Change in social cognition factor**
EF factor at pre-test	−0.457**	0.031
Social cognition factor at pre-test	−0.009	−0.535**
Evolution of EF factor	–	0.019
Age	−0.016	−0.142
School year	0.019	0.070
Attendance	−0.049	−0.059
Involvement of teacher	0.050	0.089
Number of years of service	0.121	−0.067
Mothers’ level of education	−0.13	−0.03
Fathers’ level of education	−0.11	−0.10
Family incomes	−0.07	−0.07

**p<0.001.

Thus, the lower the level of EF performance before the intervention, the more it improves after the intervention. Similarly, the weaker social cognition skills are before the intervention, the more they improve after the intervention. However, the other correlations are non-significant, which means that the change in EF and social cognition does not depend on the age and school year of the children, the number of sessions they attended, or the intensity of their teacher’s involvement in the project or their number of years of service.

## Discussion

It is now established that inhibition (Volckaert and Noël, 2015; Diamond and Ling, 2016 for a review) and social cognition skills (Houssa et al., 2013 for a review) can be improved through training. To our knowledge, there is no data in the literature concerning the potential benefit of a training program focusing on both inhibition and social cognition abilities for young typically developing children. Therefore, the innovation of the present study was to develop an intervention program aiming to stimulate typically developing young children’s inhibition and social cognition capacities. But also, to implement it in the school setting and to evaluate its direct impact on both inhibition and social cognition competences, but also on other EFs, as well as its indirect impact on academic learning. Currently, teachers need to have at their disposal pedagogical activities in a coherent and efficient program, applicable easily in their classroom of preschoolers or children at the beginning of primary school. This study therefore responds to a growing demand in kindergarten and primary classes.

Our results showed that the program was effective at improving inhibition and other EFs. A significant effect of training was observed in the selective visual attention task (Face Cancelation), in one of three inhibition tasks (Tongue Task) as well as in one of the two flexibility tasks (DCCS). After the intervention, the children thus showed a better ability to focus attention. This may perhaps facilitate entry into learning to write and read because, thanks to better attention skills, the child will be able to focus on relevant cues to solve a task, correctly discriminate letters, etc. Good attention skills will also help to better detect facial expressions of others in social situations. We also showed better inhibition capacities to control themselves and to prevent a predominant behavior. We have seen that inhibition capacities are crucial in any school and social situation. For example, having good inhibition capacities allows children to be less impulsive; be able to wait your turn; to take the time to think, to read or listen to instructions before acting; to reread yourself and check your answers before returning your sheet; to raise your hand and wait to speak before answering aloud, not to interrupt; to be more patient in conflictual or frustrating situations. So many essential skills to develop as a future adult. Finally, we highlighted that children were more able to switch between mental processes, in other words to disengage from a process to engage in another one. These flexibility capacities make it possible, for example, to make transitions easier and more fluid between two activities. They can also facilitate changes in arithmetic operation within a mathematical exercise, as well as allowing children to consider different alternatives to an obstacle or problem solving in social interaction or at school level. All these results corroborate previous findings suggesting that it is possible to enhance young children’s executive abilities (Diamond et al., 2007; Barnett et al., 2008; Röthlisberger et al., 2012; Volckaert and Noël, 2015, 2016).

For social cognition, our intervention program led to an improvement of ToM understanding capacities and of SIP skills. Children were better able to put themselves in someone else’s place, to understand their own as well as others’ emotions, desires, thoughts and beliefs, to judge the appropriateness of a social situation, to choose an appropriate reaction in their repertoire, to develop new strategies and to resolve a critical social situation (e.g., quarrel), notably because they have expanded their repertoire of adapted strategies to apply them in social situations. Socio-cognitive conflicts and metacognition have probably allowed to develop ToM and SIP skills. Indeed, the use of metacognition allows notably the child to mark a pause in order to take into account other’s point of view, generated by the socio-cognitive conflict.

In summary, as for EF abilities, our results showed that young children’s social cognition skills can be improved through training (Domitrovich et al., 2007; Merrell et al., 2008; Houssa et al., 2013; Houssa and Nader-Grosbois, 2015). In reference to links emphasized in literature between EF and SEC (Kloo and Perner, 2003) after our intervention, children are more able to inhibit their own perspective to be able to put themselves in someone else’s perspective Furthermore, children have better understanding of what induces emotions (causes of emotions) and how to react by alternative behaviors, notably by using inhibition in order to control inappropriate behavior or by putting attention on relevant and positive aspects of social situations.

A marginal transfer effect was also observed on the task assessing the understanding of the principle of number conservation. However, the intervention program did not impact the other academic learning tasks. It is worth noting that the only academic learning task showing a sensitivity to intervention was the only one involving an aspect of inhibition. To succeed in this task, the child has to inhibit the perceptive aspect of the tokens (the amount of space they occupy) in order to use a numerical strategy. These weak results in terms of academic performance are not in line with the literature, which shows that higher EF and SEC are associated with better performance at school (Gumora and Arsenio, 2002; Blair and Diamond, 2008). However, to our knowledge, there is no data in the literature showing that a training program focusing on inhibition and/or social cognition alone can enhance academic performance, so it is possible that in order to impact literacy and mathematics skills, the intervention would need to tackle these competences. For instance, an EF training program has been shown to produce larger effects when it is implemented in the school curriculum (Bodrova and Leong, 2006; Blair and Raver, 2014) than when it is used as an add-on to the existing curriculum (Bodrova and Leong, 2006; Clements et al., 2012). In addition, it has been found that math and reading abilities can be improved when children have been taught how to use EF strategies in these disciplines (Naglieri and Johnson, 2000; Iseman and Naglieri, 2011).

Finally, our results showed that the weaker a child’s EF are at the beginning of the school year, the more they improve after the intervention. In the same way, the lower their social cognition skills are at pre-test, the greater the improvement in SEC. This finding highlights the effectiveness of such a program in terms of prevention. We were thus able to draw up a “learner profile”: the children who benefited the most from the intervention were those with the weakest performance prior to it, regardless of their age, their school year, the number of sessions they attended, the involvement of the child’s teacher in the project or their number of years of service. It could be interesting to analyze if the children benefiting the most from the program are children whose families speak more about mental states, or have structuring educational practices, or even better language skills in understanding and expression.

Two results may seem challenging: the lack of impact of the number of sessions attended by the child and of the level of involvement of the teacher in the project. However, these results can be interpreted as follows: most children attended at least 16 out of 18 sessions and therefore participated in the majority of EF and SEC activities. In addition, as part of this research, an external experimenter conducted all the sessions to ensure the proper implementation of the intervention; thus, regardless of teacher involvement, children received a minimum level of stimulation. Teacher involvement was in most of the cases moderate to high. Therefore it was sufficient for this partnership, so that children benefited from the intervention.

The limitations of the study should be addressed. First, the control group was engaged in the usual classroom activities and did not receive any other intervention, which may have biased our results. Also, although we tried to reduce the variability in terms of teacher involvement by suggesting activities to complete and tools and concepts to use during the week, not all teachers showed the same level of involvement, which is very likely to have impacted our results. Secondly, despite our request to the teachers of the control group not to implement new activities stimulating social cognition or executive functions during the year, most of them did in fact discuss emotions and conflict resolution and used games/activities tackling inhibition in the classroom. Thirdly, ideally, or in a future study, half of the children in each class should be randomly selected to be part of the experimental group. This has already been done in previous studies (Houssa and Nader-Grosbois, 2015; Volckaert and Noël, 2015) but in the present study the idea was to intervene in the classes to also train teachers in care and encourage them to use the tools even in our absence. In a later study, it would be interesting to go further, train teachers and see if their training has a beneficial effect on the children in their class. Finally, another limitation concerns our baseline in which we did not measure the WM. It would have been interesting to see if our intervention had a positive effect on this, which could eventually mediate some of the effects obtained.

Our intervention program had a positive impact on EF, with higher selective attention, inhibition and flexibility capacities, and on social cognition, with higher ToM understanding and SIP capacities. Moreover, a slight transfer effect was observed in a numerical task requiring inhibition skills. In other words, we observe that the transition from one activity to another in the classroom is easier. These children have a better understanding of emotions and take better other peers’ perspective, which allows them to react in a more adapted way when they are confronted with a situation and helps them to better regulate their emotions. We also highlighted that teachers take more account of the child’s point of view. This program is then a prevention and intervention tool, which meets the demand of many teachers as mentioned above. Furthermore, these results are encouraging because they show that teachers could easily implement activities that enhance young children’s EF and SEC, and especially those of the children who need it most. Although activities are already implemented by teachers in kindergarten, it is important to optimize their effectiveness with reference to a program with evidence-based positive effects.

As previous studies have shown that it is possible to improve atypically developing children’s EF (Halperin et al., 1999; Tamm et al., 2013 for children with ADHD) and social cognition (ToM and SIP) (Jacobs and Nader-Grosbois, 2020a,b for children with intellectual disabilities) with intervention programs carried out in small groups, it would be interesting for future studies to implement a program similar to that of the present study (i.e., combining EF and social cognition in the classroom) in specialized schools. Moreover, to maximize the effects of intervention, it would be worth training the teachers so that they can implement the intervention themselves throughout the year.

To conclude, by providing teachers with a framework of activities and effective techniques to teach children to manage their agitation and impulsivity (i.e., metacognition, encourage to appeal the socio-cognitive conflicts when they have to resolve a quarrel,…), to regulate their behavior, to perceive that one child’s point of view may differ from another, it is possible to consolidate some acquired skills and to reinforce their efficacy.

## Data Availability Statement

The data analyzed in this study is subject to the following licenses/restrictions: According to the consent obtained for the present study, only the researchers, on the supervision of the project promotors, can have access to the anonymized database. Requests to access these datasets should be directed to NN-G, nathalie.nader@uclouvain.be.

## Ethics Statement

The studies involving human participants were reviewed and approved by the Ethics committee of the Psychological Sciences Research Institute. Written informed consent to participate in this study was provided by the participants’ legal guardian/next of kin.

## Author Contributions

NH conducted the experiment, analyzed the data, and wrote the manuscript. MH and AV conducted the experiment, analyzed the data, and contributed to the writing of the manuscript. M-PN and NN-G supervised the whole study and contributed to the writing of the manuscript. All the authors contributed to the article and approved the submitted version.

## Conflict of Interest

The authors declare that the research was conducted in the absence of any commercial or financial relationships that could be construed as a potential conflict of interest.
